# Intra-abdominal pregnancy complicated by uterine rupture: a case report

**DOI:** 10.1186/s12245-025-01081-1

**Published:** 2025-11-28

**Authors:** Hassan Nur Mohamud, Mohamed Jayte, Iqra Abdullahi Mohamed, Inzama Wilfred, Abdullahi Mohamed Hussein, Okelo James

**Affiliations:** 1 Obstetrics and Gynecology Department at Kampala International University, P.O. Box 7062, Kampala, Uganda; 2 Internal Medicine Department at Kampala International University, Kampala, Uganda

**Keywords:** Intra-abdominal pregnancy, Uterine rupture, Ectopic pregnancy, Advanced gestation, Emergency laparotomy, Maternal outcome, Uganda

## Abstract

**Background:**

Intra-abdominal pregnancy is a rare and life-threatening form of ectopic gestation that poses significant diagnostic and management challenges, particularly in low-resource settings. Its occurrence alongside uterine rupture further increases maternal risk.

**Case presentation:**

We report the case of a 22-year-old gravida 4, para 1 + 2 Ugandan woman at 28 weeks + 3 days of gestation who presented with lower abdominal pain and vaginal bleeding. She had not attended antenatal care or undergone prior imaging. On examination, fetal heart sounds were absent, and the uterus was tender with smaller-than-expected fundal height. A provisional diagnosis of intra-abdominal pregnancy with possible uterine rupture was made. Emergency laparotomy revealed a ruptured uterine fundus with an intact gestational sac containing a macerated stillborn male fetus weighing 1.2 kg. The necrotic tissue was excised, and the uterine defect was repaired in layers. The postoperative course was uneventful, and the patient was discharged in stable condition.

**Conclusion:**

This case highlights the critical need for early antenatal attendance, access to basic obstetric ultrasound, and timely referral to avert complications associated with advanced extra-uterine pregnancies and uterine rupture. Strengthening maternal health systems and clinical suspicion remains vital to improving outcomes in similar low-resource settings.

## Introduction

Intra-abdominal pregnancy, a rare form of ectopic gestation wherein the embryo implants within the peritoneal cavity outside the uterine corpus, fallopian tubes, or ovaries, presents unique diagnostic and therapeutic challenges. Globally, ectopic pregnancies account for approximately 1%–2% of all pregnancies, but abdominal pregnancies comprise a much smaller fraction—estimated at 1:10,000 to 1:30,000 pregnancies [1]. Despite its rarity, when it occurs, it is associated with high maternal and perinatal morbidity and mortality due to delayed diagnosis, atypical presentation, and often complex surgical management.

In sub-Saharan Africa, obstetric emergencies such as uterine rupture and ectopic gestations remain significant contributors to maternal mortality. The incidence of uterine rupture in low-income settings may reach 1.3% or higher, reflecting delays in seeking care, limited antenatal attendance, and suboptimal referral systems [2]. In Uganda specifically, studies have reported an incidence of uterine rupture as high as 1 in 131 deliveries (0.76%) in one regional referral hospital [3]. Such figures highlight ongoing challenges in maternal health services, including inadequate labour monitoring, accessibility issues, and delays in transfer to capable facilities.

The problem remains that, although broad obstetric indicators (e.g., antenatal coverage) may have improved, rare but catastrophic events like advanced abdominal pregnancy or uterine rupture often escape early detection in low-resource settings. In Uganda, one study found a ratio of uterine rupture at 1 in 200 deliveries in a rural district [4]. These complications continue to reflect gaps in timely obstetric care, referral linkage, ultrasound availability, and skilled surgical response.

This case is therefore reported because it combines two high-risk obstetric conditions — an intra-abdominal pregnancy **and** a ruptured uterus — occurring in a resource-limited setting. The unusual presentation underscores the need for high clinical suspicion, robust referral pathways, and readiness for emergency laparotomy in settings like Uganda. In addition, documenting such a case contributes to the limited literature from sub-Saharan Africa on intra-abdominal pregnancies reaching advanced gestation, and adds practical insight for clinicians managing rare obstetric emergencies.

## Case presentation

A 22-year-old Ugandan female, gravida 4 para 1 + 2, residing in Apac District, was referred to Lira Regional Referral Hospital with a 7-day history of lower abdominal pain and intermittent bright-red per-vaginal bleeding. The pain was gradual in onset, radiating to the back and progressively worsening despite analgesic use. It was associated with nausea, fatigue and dizziness, but there was no history of fever, urinary complaints, vomiting or gastrointestinal symptoms.

The pregnancy was unplanned, and she had not attended any antenatal care. Her last menstrual period (LMP) was patient-estimated as 24th March 2024, corresponding to a gestational age of 28 weeks + 3 days at presentation. She reported becoming aware of the pregnancy only around the fifth month of gestation and had noticed minimal abdominal growth during the preceding week.

Prior to referral, an obstetric ultrasound performed at the general hospital showed an empty uterus with a viable fetus visualized superior to the uterine fundus, raising suspicion of an extra-uterine gestation. Based on these findings and her persistent abdominal pain, she was transferred for further evaluation and emergency management.

Her obstetric history revealed four pregnancies. The first pregnancy in January 2021 ended in a spontaneous abortion at three months without complications. The second pregnancy, in November 2021, resulted in a normal vaginal delivery of a healthy male infant at term at a Health Centre III. The third pregnancy, in November 2023, ended in a second-trimester abortion at five months, followed by a uterine evacuation (dilatation and curettage) without subsequent complications. The current (fourth) pregnancy was unplanned, and she had no history of contraception use.

She had no known chronic medical conditions such as hypertension, diabetes mellitus, or heart disease. Her surgical history was notable only for the prior evacuation procedure in 2023. There was no family history of medical disorders, and she did not smoke or consume alcohol. Her husband was a farmer and likewise did not smoke or drink.

On general examination, she appeared conscious and alert, with mild pallor and no jaundice, edema, or dehydration. Vital signs were stable with blood pressure at 121/78 mmHg, pulse rate 90 bpm, temperature 35.0 °C, and oxygen saturation of 99% on room air. Abdominal examination revealed movement with respiration and generalized tenderness, most marked in the lower abdomen. The abdomen was soft, and fetal parts were palpable; however, fetal heart sounds were not detected. The symphysiofundal height measured 26 cm, which was smaller than the expected gestational age. Vaginal examination revealed a warm, moist vagina with blood-stained secretions. On speculum assessment, the cervix was closed with no active bleeding. Bimanual examination elicited marked cervical motion tenderness.

A provisional diagnosis of intra-abdominal pregnancy with possible uterine rupture was made. The differential diagnoses included advanced ectopic gestation, concealed abruptio placentae, and ruptured uterus secondary to neglected intrauterine pregnancy. The patient was admitted to the gynecology ward for stabilization and further evaluation.

Pre-operative management included intravenous (IV) fluid resuscitation with normal saline (2 L) and dextrose 5% (1 L). Laboratory investigations such as complete blood count, blood grouping, and cross-matching were performed, and two units of blood were reserved. Empirical IV ampicillin 2 g was initiated, and the theatre and anesthesia teams were informed. A urinary catheter was inserted for monitoring urine output. Consent for emergency laparotomy was obtained from the patient’s next of kin after adequate counseling.

### Operative findings and procedure

**Fig. 1:**
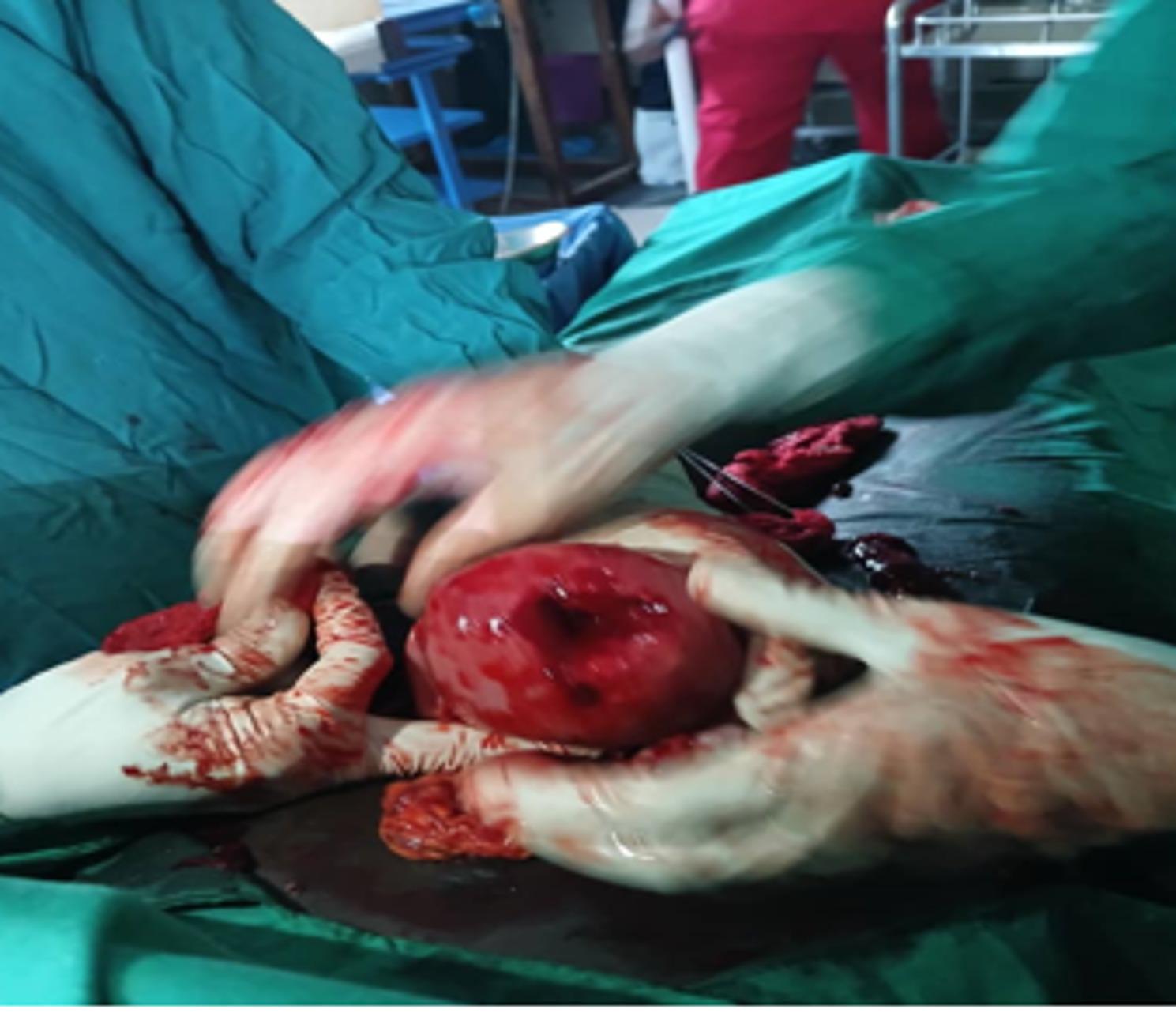
Showing uterus appeared ruptured at the fundus

**Fig. 2:**
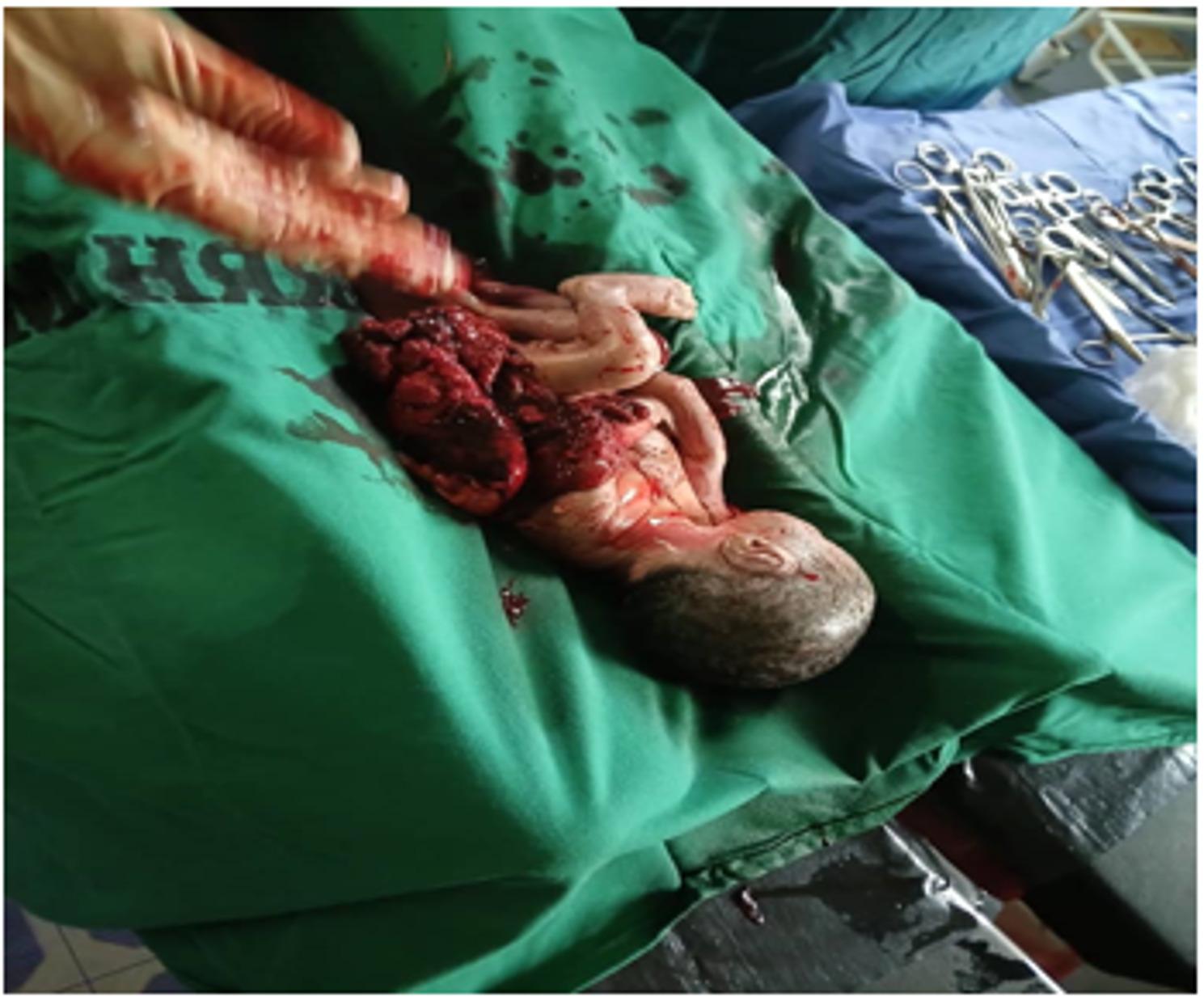
Showing macerated stillborn male fetus weighing 1.2 kg

Under aseptic precautions and spinal anesthesia, a midline sub-umbilical incision was made. Upon entering the peritoneal cavity, approximately 200 mL of altered blood was observed. The uterus appeared ruptured at the fundus (Fig. 1), with an intact gestational sac containing a macerated stillborn male fetus weighing 1.2 kg (Fig. 2). The placenta was partially attached to the uterine serosa and the omentum, confirming an advanced intra-abdominal pregnancy with secondary rupture of the uterine fundus. The rupture edges were fibrotic, suggesting a chronic process rather than an acute tear. The necrotic tissue was excised, and the uterine defect was repaired in two layers using absorbable Vicryl 2 − 0 sutures. Hemostasis was meticulously achieved. The rectus sheath was closed with Vicryl 1, and the skin was approximated using nylon 1 sutures. The surgical wound was covered with sterile gauze and plaster dressing.

### Post-operative management

The patient was closely monitored in the recovery unit for vital signs—blood pressure, pulse, and temperature every 15 min for the first hour, every 30 min for two hours, and then hourly for 24 h. She was maintained on IV normal saline 2 L and dextrose 5% 1 L every six hours for 24 h. IV piperacillin-tazobactam 4.5 g once daily and IV metronidazole 500 mg three times daily were continued for three days to prevent infection. Analgesia included IV paracetamol 1 g three times daily and intramuscular diclofenac 75 mg three times daily for two days. She remained nil per os (NPO) for six hours post-operation and continued to be observed for PV bleeding. Antibiotic selection was empirical and influenced by the likely polymicrobial risk from intra-abdominal contamination and partial placental attachment to the omentum/serosa. Pre-operative IV ampicillin was given; post-operatively the patient received broad-spectrum IV piperacillin-tazobactam and metronidazole to cover gram-negative bacilli and anaerobes, guided by local formulary availability and standard surgical prophylaxis practice in our setting. No intra-operative cultures were obtained [5].

On the first post-operative day, she reported only mild incisional pain. She remained afebrile, and her vital signs were stable (BP 121/69 mmHg, PR 79 bpm, SpO₂ 99%). Abdominal examination revealed a soft abdomen with a clean, dry incision. Vaginal examination demonstrated normal lochia rubra. On the second post-operative day, she was comfortable, and the wound remained clean with no signs of infection. Oral intake was resumed, and she continued antibiotics as prescribed. The patient was counselled to avoid conception for at least 12 months to allow uterine healing and to use reliable contraception. Future pregnancies will be high-risk: early antenatal booking, serial ultrasound assessment of uterine integrity and placental location, and delivery in a tertiary facility with readiness for operative delivery and blood transfusion are recommended. Mode of delivery in future pregnancies should be individualized; an elective cesarean section may be advised if scarring or weakened myometrium is suspected [6].

### Follow-up

The patient was reviewed regularly in the gynecology ward for wound care, anemia assessment, and counseling regarding future pregnancies. She was advised to delay conception for at least one year and to attend antenatal care early in any subsequent pregnancy to minimize the risk of recurrence or complications.

## Discussion

Our case describes a 22-year-old woman who presented at 28 weeks + 3 days with seven days of lower abdominal pain and intermittent bright red vaginal bleeding. On arrival she had a smaller-than-expected fundal height, palpable fetal parts with absent fetal heart tones, and clinical signs suggesting an extra-uterine advanced gestation complicated by rupture of the uterine fundus. Emergency exploratory laparotomy confirmed a secondary abdominal pregnancy with a macerated stillborn (1.2 kg) and a ruptured uterine fundus; the rupture edges appeared old and fibrotic. The patient underwent excision of necrotic tissue and layered uterine repair with uneventful early postoperative recovery.

This presentation aligns with the typical clinical pattern described for abdominal pregnancy: nonspecific abdominal pain, abnormal fetal size or presentation, and difficulty in detecting fetal heart sounds, often in women who have had little or no antenatal imaging [1]. Abdominal pregnancies are rare but clinically important because they commonly escape early detection; series and reviews show that a substantial proportion (often >70%) of abdominal pregnancies are not diagnosed until surgery or advanced gestation, particularly in low-resource settings where routine ultrasound is limited [1, 2]. Reported maternal outcomes vary widely depending on timing of diagnosis and the site of placental implantation, but perinatal survival is generally poor when diagnosis is delayed—many reported series note fetal demise in a majority of advanced cases [3, 4].

The occurrence of uterine rupture in this patient is also consistent with patterns seen in sub-Saharan Africa, where ruptured uterus remains a major cause of maternal morbidity and mortality. Case series from Uganda and similar settings report uterine rupture incidence rates and a substantial case fatality ratio, linked to delays in seeking care, obstructed labour, previous uterine instrumentation, and inadequate antenatal follow-up [7, 8]. In our patient, a history of a second-trimester uterine evacuation in 2023 may have contributed to uterine wall weakness; prior surgical instrumentation is a recognized predisposing factor for uterine dehiscence or rupture, especially if a subsequent pregnancy is unmonitored [7, 8]. In many African reports, delayed presentation and poor access to ultrasound or timely referral are repeatedly implicated as roots of adverse outcomes—factors that were evident in this case where antenatal care had been absent and the pregnancy was first recognized late [7, 9].

Management decisions in advanced abdominal pregnancy are shaped chiefly by hemodynamic stability, placental implantation, fetal viability, and available surgical expertise. The literature supports immediate laparotomy when maternal condition is unstable or when there is suspicion of rupture or hemorrhage; conservative expectant management until fetal maturity has been described only when diagnosis is made early and close monitoring is feasible [10–12]. Intra-operative handling of the placenta is a critical determinant of maternal risk: attempts to remove a placenta that is extensively adherent to pelvic or abdominal viscera can precipitate life-threatening hemorrhage, whereas leaving the placenta in situ risks infection, abscess or secondary hemorrhage and may require prolonged follow-up or adjuvant therapies [2, 3, 10]. In our patient the placenta was partially attached to the uterine serosa and omentum; the surgical team excised necrotic tissue and repaired the uterine defect while achieving hemostasis—an approach consistent with reports that, when feasible and safe, limited resection and repair with careful control of bleeding can yield good maternal outcomes [1, 13]. When abdominal pregnancy is diagnosed early, termination (surgical or, in selected early cases, medical) is appropriate. In advanced abdominal pregnancy, expectant management until fetal maturity has been reported but requires close surveillance, repeated imaging, blood bank availability and multidisciplinary care — conditions often unavailable in low-resource settings. Removal of placenta risks massive haemorrhage; leaving the placenta in situ is an accepted strategy when placental implantation is extensive, albeit with risk of infection or delayed hemorrhage. Use of methotrexate and arterial embolization has been described as adjuncts in selected cases but are not universally available and carry their own risks [14].

Fetal maceration and intrauterine fetal demise in advanced extra-uterine gestation are common findings; several series document preoperative fetal death in most advanced abdominal pregnancies, particularly when diagnosis is late [15, 16]. Fetal death itself carries maternal risks, including coagulopathy in some settings; studies relating fetal maceration grade to maternal coagulation abnormalities underscore that clinicians should monitor for hemostatic derangements after removal of a macerated fetus and placental tissue [15]. In our patient there were no immediate coagulopathic signs and hemostasis was achieved intra-operatively, but the risk of late hemorrhage or infection remains a recognized concern requiring prolonged follow-up in other reported cases [2, 10].

Comparing our case to published African series and case reports, the pattern is sadly familiar: late recognition, lack of antenatal imaging, prior uterine procedures, and emergency laparotomy with variable placental involvement. Some African case reports document maternal survival with conservative placental management or staged removal and intensive supportive care, while other series stress high rates of fetal loss and significant maternal morbidity when rupture or massive hemorrhage occurs [3, 4, 9]. Conversely, isolated reports from higher-resource centres document favourable fetal outcomes when abdominal pregnancy is diagnosed early and managed in a controlled, multidisciplinary setting—emphasizing the critical role of early ultrasound and referral pathways [3, 10].

Common implantation sites described include the pouch of Douglas, omentum, uterine serosa (including fundal serosa), adnexa, pelvic peritoneum and, rarely, visceral organs such as bowel, liver or spleen. Secondary abdominal pregnancies with partial placental attachment to uterine serosa and omentum — with subsequent uterine rupture — have been reported in case series and reviews, particularly from low-resource settings where antenatal ultrasound is limited [14].

There is no single universally accepted guideline for management of abdominal pregnancy. Consensus from reviews and specialty guidance (e.g., ISUOG and contemporary literature) recommends individualized management depending on gestational age, placental attachment, and maternal stability, with multidisciplinary planning for advanced cases [17].

This case highlights several practical lessons. First, clinicians working in low-resource settings should maintain a high index of suspicion for extra-uterine advanced pregnancies when fundal height and fetal heart findings are discordant with gestational age or when vaginal bleeding and abdominal pain occur late in pregnancy without clear cause. Second, improving access to basic obstetric ultrasound and strengthening antenatal attendance could enable earlier diagnosis and planned management, potentially reducing the need for emergency laparotomy and improving perinatal outcomes. Third, when surgery is required, careful intra-operative decision-making about placental management and meticulous haemostasis are paramount; readiness for blood transfusion and postoperative monitoring for infection and coagulopathy are essential.

## Conclusion

This case demonstrates a rare obstetric emergency—an advanced intra-abdominal pregnancy complicated by uterine rupture—presenting late in a setting of absent antenatal care and unavailable imaging. Timely surgical intervention achieved a favourable maternal outcome despite fetal loss. The case underlines the importance of early antenatal booking, access to basic obstetric ultrasound, strengthened referral pathways and preparedness for emergency laparotomy in resource-limited settings. Conclusions are limited by this being a single case with incomplete preoperative imaging.

## Data Availability

Not applicable.
